# Altered intrinsic neural activity and its molecular analyses in first-episode schizophrenia with auditory verbal hallucinations

**DOI:** 10.3389/fnins.2024.1478963

**Published:** 2024-10-29

**Authors:** Ziyu Wang, Kangkang Xue, Yimeng Kang, Zijun Liu, Jingliang Cheng, Yan Zhang, Yarui Wei

**Affiliations:** ^1^Department of Magnetic Resonance Imaging, the First Affiliated Hospital of Zhengzhou University, Zhengzhou, China; ^2^Key Laboratory for Functional Magnetic Resonance Imaging and Molecular Imaging of Henan Province, Zhengzhou, China

**Keywords:** schizophrenia, auditory verbal hallucinations, amplitude of low-frequency fluctuation, neurotransmitter, intrinsic neural activity

## Abstract

**Background:**

Auditory verbal hallucinations (AVHs) are one of the signature positive symptoms of schizophrenia, affecting a substantial portion of patients with schizophrenia. These hallucinations seriously impact the lives of patients, resulting in a substantial social burden. Recent studies have shown a significant correlation between abnormal local brain activity and the neurobiological mechanisms of AVHs. However, it is not fully clear whether altered intrinsic brain activity in schizophrenia patients with AVHs is correlated with specific neurotransmitter systems.

**Methods:**

We included 50 first-episode, drug-naïve schizophrenia patients with AVHs, 50 patients without AVHs (NAVHs), and 50 age- and sex-matched healthy controls (HCs). The amplitude of low-frequency fluctuation (ALFF) was utilized to explore the altered intrinsic brain activity in the AVH group. Subsequently, we spatially correlated the altered ALFF with neurotransmitter maps using JuSpace.

**Results:**

In our study, compared to HCs, the AVH group exhibited significantly reduced ALFF in multiple brain regions, mainly including the left precuneus, bilateral supplementary motor areas, bilateral paracentral lobules, bilateral precentral gyri, and bilateral postcentral gyri. The NAVH group showed significantly reduced ALFF in the left inferior occipital gyrus, left calcarine gyrus, and left lingual gyrus compared to HCs. Furthermore, the AVH group showed higher ALFF in the right inferior frontal gyrus compared to the NAVH group. Additionally, these ALFF alterations in the AVH group were closely related to three neurotransmitters, including dopamine, serotonin and norepinephrine.

**Conclusion:**

We link neurotransmitters to abnormal intrinsic brain activity in first-episode, drug-naïve schizophrenia patients with AVHs, contributing to a comprehensive understanding of the pathophysiological processes and treatment pathways underlying AVHs.

## Background

Schizophrenia is a complex neuropsychiatric disorder characterized by a spectrum of cognitive, behavioral, and emotional dysfunctions (Faden and Citrome, 2023). Currently, the diagnosis of schizophrenia relies on a comprehensive clinical assessment, usually made by a professional psychiatrist based on a set of criteria outlined in the Diagnostic and Statistical Manual of Mental Disorders, Fourth Edition (DSM-IV) (Tandon et al., 2013). Among all symptoms, auditory verbal hallucinations (AVHs), defined as the perceived experience of sound that occurs in the absence of any corresponding external stimulus, are one of the core symptoms of schizophrenia, affecting approximately 60–90% of patients with schizophrenia (Bauer et al., 2011) and nearly 30% of patients with AVHs respond poorly to medication (Alderson-Day et al., 2015). Most of the symptoms of hallucinations are not controlled by patients, which is an important factor leading to mental disability and even self-injury and other injuries. Understanding the neurobiological mechanisms involved in schizophrenia with AVHs can help further elucidate the pathophysiological basis of AVHs and provide an objective theoretical basis for achieving accurate medical treatments, ultimately alleviating the suffering of patients and improving their quality of life.

Over time, the following several theoretical models have been proposed as researchers employ different techniques to explore the etiology of AVHs. First, the self-monitoring of inner speech model, proposed by Frith and Done (1988), indicates that failures in self-monitoring can lead to internally generated activities, such as inner speech, being mistaken for externally generated sounds, thereby producing the symptoms of AVHs (Wolpert et al., 1995; Frith et al., 1992). Second, Dierks et al. (1999) introduced the spontaneous neural activity model and found when comparing the resting state activity during AVHs with resting state activity without AVHs in the same patients, hallucination-related activities were observed in Broca’s area, temporal gyrus, and primary auditory cortex. Based on this finding, successive researchers (Ford et al., 2009; Hunter et al., 2006) have proposed that AVHs may be due to an abnormally increased resting state activity in the auditory cortex. Ford and Hoffman (Ford and Hoffman, 2013) later suggested that the self-monitoring model and the spontaneous neural activity model are not mutually exclusive, and proposed a hybrid model combining the two. In recent years, with the in-depth research of functional magnetic resonance imaging (fMRI), diffusion tensor imaging (DTI), and electroencephalography (EEG), some researchers believe that the abnormality of the interhemispheric auditory pathway may be the pathological mechanism of AVHs (Steinmann et al., 2019; Gavrilescu et al., 2010; Hubl et al., 2004). Each hypothesis regarding the neural mechanisms of AVHs may involve underlying abnormal brain activity. In addition, schizophrenia is thought to be associated with dynamic abnormalities in the brain that are initiated during the early stages of brain development (Pino et al., 2014; Rapoport et al., 2012). Therefore, assessing altered intrinsic brain activity may help elucidate the underlying pathophysiological mechanisms associated with the development of AVHs in patients with schizophrenia. Spontaneous neural activity under resting state is crucial for understanding neuropathological and neurophysiological conditions (Fox and Raichle, 2007). As a reliable and non-invasive method, resting state functional magnetic resonance imaging (rs-fMRI) has gained popularity due to its ease of implementation and ability to avoid potential task-related confounds, making it an increasingly commonly used approach for studying neuropsychiatric disorders. The amplitude of low-frequency fluctuation (ALFF) (Zang et al., 2007) is a frequently used index for quantifying the intensity of intrinsic brain activity based on rs-fMRI data and is widely applied in clinical studies. Although ALFF has been extensively utilized in research involving patients with schizophrenia and HCs, fewer studies have focused on the ALFF alterations between patients with AVHs and healthy individuals. For example, a study investigating the role of the putamen in schizophrenia patients with AVHs reported increased ALFF in the putamen in first-episode schizophrenia with AVHs (Cui et al., 2016). Similarly, Alonso-Solís et al. (2017) identified a specific pattern of ALFF alterations in the insula and medial temporal regions in schizophrenia patients, both with and without persistent AVHs. Moreover, a study exploring the correlation between hallucination severity and ALFF found that altered ALFF in the left hippocampus may play a pivotal role in the onset and prolonged manifestation of AVHs (Hare et al., 2017). These studies suggest that altered ALFF dynamics may represent a neurobiological basis for AVHs. Additionally, ALFF measures the absolute amplitude of low-frequency fluctuations, providing a direct reflection of the overall intensity of spontaneous brain activity. Given that AVHs are a prominent and impactful symptom in schizophrenia, capturing this absolute intensity in relevant brain regions is essential for understanding their underlying mechanisms. Therefore, exploring alterations in ALFF values among schizophrenia patients with AVHs is also necessary to deepen our understanding of the neurobiological mechanisms underlying AVHs.

Each time a neuron is activated in brain activity, whether due to spontaneous fluctuations or neural activation induced by external stimulation, it eventually manifests in corresponding changes at the level of receptors and neurotransmitters, resulting in changes in the function or structure of the brain at the macro level (Stanfield, 2013). Excitatory-to-inhibitory (E/I) balance is necessary for optimal neural signal formation, synchrony, and transmission, which, in turn, support information processing driving both simple and complex behaviors (Yizhar et al., 2011). Currently, an imbalance between excitatory and inhibitory influences (E/I imbalance) in the brain is proposed as a potential mechanism underlying the etiology of auditory verbal hallucinations (AVHs) (Hjelmervik et al., 2020; Hugdahl, 2015; Jardri et al., 2016), existing on multiple levels, including small-scale neuronal circuits (within regions) and large-scale cognitive networks (between regions). Evidence suggests that an imbalance in glutamate (Glu) and gamma-aminobutyric acid (GABA) levels within language and cognitive control regions of the bilateral frontotemporal network, including the speech perception area (superior temporal gyrus [STG]) (Kompus et al., 2011) and speech production area (Broca’s area in the inferior frontal gyrus [IFG]) (Kuhn and Gallinat, 2012), is thought to mediate the development of AVHs in schizophrenia patients (Hugdahl, 2015; Ćurc̆ić-Blake et al., 2017a). Studies involving ketamine, which blocks glutamate from binding to GABAergic neurons, have shown that the drug exacerbates psychotic symptoms such as hallucinations in schizophrenia patients (Lahti et al., 2001) and induces schizophrenia-like symptoms in healthy subjects (Lahti et al., 2001; Abram et al., 2022; Thiebes et al., 2018). The interhemispheric miscommunication theory of AVHs posits that aberrant connectivity between the left and right superior temporal gyrus (STG) plays a critical role in the occurrence of AVHs (Mulert et al., 2011; Steinmann et al., 2014). This connectivity is notably modulated by Glu and GABA, indicating that neurochemical imbalances significantly contribute to the disruptions in interhemispheric STG functional connectivity (Egerton et al., 2017; Hugdahl et al., 2015). Further research indicates that NMDA receptor dysfunction in the left STG may lead to neuronal hyperactivity and elevated levels of Glu and Glx (Glu+glutamine), resulting in the perceptual experience of “hearing voices” (Nudmamud and Reynolds, 2001). In a normally functioning brain, the anterior cingulate cortex (ACC) would be actively engaged to suppress or inhibit these “voices” from reaching awareness. However, in a hallucinating brain, this top-down regulation might be upset due to low Glx levels in the ACC, together with, or as a consequence of, “strong” bottom-up perceptual impulses, resulting in AVH (Hjelmervik et al., 2020; Hugdahl, 2015; Ćurc̆ić-Blake et al., 2017b; Hugdahl, 2009). Sarah Weber and colleagues recently found that Glx and GABA concentration levels in the ACC demonstrated significant relationships with interhemispheric STG connectivity. Specifically, Glu levels showed a positive association, while GABA levels had a negative association with STG connectivity (Weber et al., 2021). To gain a deeper understanding of auditory verbal hallucinations (AVHs) in schizophrenia, disturbances in the neurotransmitter system are an essential factor. Currently, antipsychotic medications primarily target the neurotransmitter system to reduce the frequency and severity of hallucinations in schizophrenia patients (Sommer et al., 2012). Therefore, imbalances in the neurotransmitter system not only reveal the neurobiological basis of hallucinations but also provide potential targets for future therapeutic interventions. Magnetic Resonance Spectroscopy (1H-MRS) is a non-invasive imaging technique that allows the measurement of *in vivo* concentrations of various brain metabolites, providing insight into neurotransmitter system abnormalities in schizophrenia (Merritt et al., 2016). Currently, MRS is not only used to study the glutamatergic and GABAergic systems, which are crucial in understanding the neurochemical basis of schizophrenia, but also to assess metabolites such as N-acetylaspartate (NAA) and choline compounds, which are linked to neuronal integrity and membrane turnover (Bustillo et al., 2008). With continuous advancements in MRS acquisition and processing techniques, researchers have gained valuable insights into the neurobiological underpinnings of schizophrenia, including the roles of neurotransmitter imbalances and altered brain metabolism (Ongur et al., 2009). However, previous studies used voxel- or region-based analyses, leading to low-to-moderate test-retest reliabilities. Recent studies have demonstrated that the overall spatial activity patterns can provide more reliable information than standard voxel- and region-based analyses (Dukart et al., 2021; Holiga et al., 2018). In this context, a new toolbox named JuSpace, developed by Dukart et al. (2018), analyzes the spatial associations between SPECT- and PET-derived neurotransmitter information and other modality data. This toolbox has been used to explore the spatial associations between neurotransmitter distribution and brain activity, as well as gray matter volume (GMV) changes in various conditions, such as Parkinson’s disease (Shi et al., 2024), primary progressive aphasia (Premi et al., 2023a), prodromal frontotemporal dementia (Premi et al., 2023b), and schizophrenia (Chen et al., 2023). Currently, the spatial associations between the altered intrinsic brain activity and the neurotransmitter system in schizophrenia concerning AVHs remain unclear. Given that further research is still needed to explore the relationship between altered brain functions and the possible pathophysiological mechanisms underlying AVHs, the primary purpose of this study was to explore the patterns of altered intrinsic brain activity in schizophrenia patients with AVHs, applying the ALFF of rs-fMRI metrics. The second purpose was to employ the JuSpace toolbox to evaluate the patterns of neurotransmitter defects in those patients with AVHs based on imaging differences among schizophrenia patients with and without AVHs, as well as HCs. We hypothesized that: (i) distinct altered intrinsic brain activity would be observed in schizophrenia patients with AVHs, and (ii) these imaging differences showing static characteristics might be potentially associated with aberrant neurotransmitter system.

## Materials and methods

### Participants

We included 100 drug-naïve, first-episode schizophrenia (FES) patients (50 with AVHs and 50 without AVHs) and 50 age- and sex-matched healthy controls (HCs) to ensure that brain activity assessments are not influenced by medication, allowing for a more accurate evaluation of the brain’s natural functional state, particularly in early-stage AVHs. Schizophrenia was diagnosed according to DSM-IV criteria by a psychiatric specialist. The illness duration of all patients was less than 3 years, and the period since disease onset was less than 6 months. Each participant was carefully evaluated by a psychiatrist to ensure they had not received pharmacological treatment for schizophrenia or any related psychiatric condition, including anxiolytics, benzodiazepines (BDZs) or other GABAergic medication. Symptom severity of schizophrenia was assessed with the Positive and Negative Syndrome Scale (PANSS).

Within the past 4 weeks, 50 patients reported experiencing AVHs, most of them within the past week, while the other 50 patients reported no AVHs either in their lifetime or in the past 4 months. The severity of AVHs was assessed using the Auditory Hallucination Rating Scale (AHRS). We collected PANSS data for 33 of the AVH patients and for all NAVH patients, and AHRS data for all AVH patients. All participants were right-handed. Exclusion criteria for all participants were as follows: (1) contraindications for MRI, (2) alcohol or drug abuse, and (3) severe physical disability and traumatic head injuries. HCs had no history of neurological or psychiatric illness. All subjects gave the informed consent, and this study was approved by the Ethics Committee of the First Affiliated Hospital of Zhengzhou University.

### Data acquisition

All participants were scanned with a 3.0 T MRI scanner (Discovery MR750, GE, USA), which utilized an 8-channel receiver array head coil. Head motion and scanner noise were minimized with foam padding and earplugs, while participants were instructed to lie flat, close their eyes, avoid thinking of anything specific, breathe quietly, and not fall asleep. The following scanning parameters were used to acquire functional images:

TR/TE = 2,000/30 ms, number of slices = 32, slice thickness = 4 mm, slice gap = 0.5 mm, flip angle = 90°, FOV = 22 × 22 cm^2^, number of averages = 1, matrix size = 64 × 64, voxel size = 3.4375 × 3.4375 × 4 mm^3^, and 180 volumes in total. The patients in the AVH group reported that they experienced no hallucinations during scanning.

### Data preprocessing

All fMRI images were initially checked for quality, and any incomplete or artifact-filled images were excluded. Data preprocessing was performed by using Data Processing Assistant for Resting-State fMRI (DPARSFA). Several steps were involved in preprocessing: (i) removing first 5 volumes considering unsteady magnetization; (ii) slice-timing; (iii) spatially normalized to the Montreal Neurological Institute template (resampling voxel size = 3 × 3 × 3 mm^3^); (iv) smoothed using an isotropic Gaussian kernel [full width at half maximum (FWHM) = 6 mm]; (v) removing linear trends and temporally bandpass filtering (0.01– 0.08 Hz) to eliminate low-frequency drift and high frequency noise influences; (vi) regression of head motion parameters, global signals, white matter and cerebrospinal fluid signals.

### ALFF calculation

The ALFF analysis was carried out using DPARSFA software (v5.4). The preprocessed time series of each voxel was transformed into the frequency domain with a Fast Fourier Transform and the power spectrum was then obtained. We measured ALFF by obtaining the square root of the signal across 0.01–0.08 Hz for each voxel. For standardization purposes and to reduce the influence of individual variation in ALFF values, the ALFF of each voxel was further divided by the global mean of ALFF values for each subject within the default brain mask from the DPARSFA, with background and other non-brain tissue signals removed. This created a standardized whole-brain ALFF map.

### Spatial correlation with neurotransmitter density maps

The JuSpace toolbox (version 1.5) was used to evaluate if the spatial patterns of ALFF changes in schizophrenia patients with AVHs were correlated with specific neurotransmitter systems. The JuSpace toolbox furnished data on neurotransmitter receptors and transporters derived from prior PET/SPECT studies involving healthy volunteers, including the dopaminergic (dopamine D1 and D2; dopamine transporter: DAT; the dopamine synthesis capacity: PET/SPECTOPA); serotonergic system (serotonin 5-hydroxy tryptamine receptor subtypes 1a, 1b, 2a, and 4: 5-HT1a, 5-HT1b, 5-HT2a, 5-HT4; serotonin transporter: SERT); noradrenergic system (noradrenaline transporter: NAT); GABAergic system; Glutamate Receptor (GluR); μ-opioid receptor system (MU); N-methyl-D-aspartic acid receptor (NMDA); vesicular acetylcholine transporter (VAChT); and cannabinoid receptor type 1 (CB1). The default neuromorphometrics atlas was utilized, excluding all WM and CSF (Dukart et al., 2021).

### Statistical analysis

Using the statistical module in the DPABI toolkit, a one-way ANOVA was used to compare ALFF data in the AVH, NAVH and HC groups with age and sex and mean FD as covariates. Then, *post hoc* comparisons using Bonferroni’s test were performed, and pair-wise different *Z*-value maps corresponding to between-group ALFF differences were computed. The statistics were then corrected for Gaussian random fields (GRF), and *P* < 0.001 at the voxel-level and *P* < 0.05 at the cluster level were considered statistically significant.

Spearman’s correlation coefficients (Fisher’s *Z* transformed) were calculated between these ALFF maps and the spatial distribution of the respective neurotransmitter maps. Exact permutation-based *P*-values as implemented in JuSpace (10,000 permutations randomly assigning group labels using orthogonal permutations) were computed to test if the observed correlation coefficients across patients deviate from a null distribution. The false discovery rate (FDR) was used for the correction of multiple comparisons.

## Results

### Demographic and clinical data

No significant difference in age or sex was found, and no significant difference in PANSS total, positive, negative, general, or delusion scores was found between the AVH and NAVH groups, except for hallucination scores (see Table 1).

**TABLE 1:** Demographic and clinical data.

	AVH	NAVH	NC	*F*/*X*^2^/*t* Values	*P*-values
Age (SD, *n* = 50)	21.3 (7.7)	21.3 (7.7)	22 (7.7)	0.139	0.871
Sex (M/F, *n* = 50)	24/26	25/25	24/26	0.053	0.974
AHRS (SD, *n* = 50)	23.86 (5.99)	–	–	–	–
Medication History	No	No	–	–	–
**PANSS (SD, *n* = 50) (AVH: *n* = 33; NAVH: *n* = 50)**					
PANSS total	83.3 (14.6)	82.6 (15.9)	–	0.204	0.839
PANSS positive	20.3 (5.4)	19.6 (6.2)	–	0.537	0.593
PANSS negative	20.8 (4.8)	21.0 (5.6)	–	−0.128	0.899
PANSS general	42.2 (7.5)	42.0 (8.6)	–	0.077	0.939
PANSS hallucinations	4.1 (1.5)	2.4 (1.6)	–	4.073	0.0002
PANSS delusions	4.7 (1.4)	3.8 (1.7)	–	1.966	0.056

AHRS, auditory hallucination rating scale; AVH, auditory verbal hallucination; F, female; M, male; NAVH, without auditory verbal hallucination; NC, normal control; PANSS, positive and negative syndrome scale.

### ALFF group differences

**FIGURE 1:**
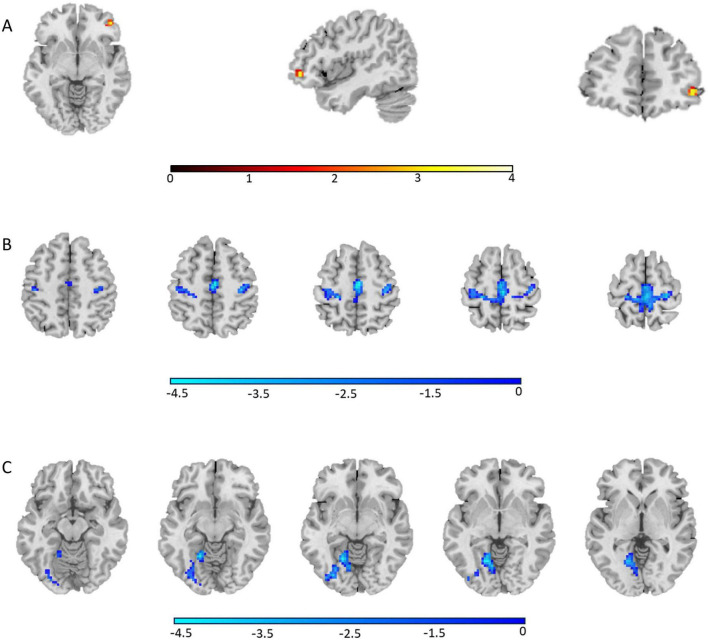
The regions where ALFF values were altered in the whole-brain analysis (one-way ANOVA, GRF corrected, *P*_voxel_ < 0.001, *P*_cluster_ < 0.05). **(A)** Group differences of ALFF between the AVH and NAVH groups; **(B)** Group differences of ALFF between the AVH and HC groups; **(C)** Group differences of ALFF between the NAVH and HC groups.

The AVH group showed significant enhanced ALFF in the right inferior frontal gyrus compared to the NAVH group (see Figure 1A and Table 2), and reduced ALFF in the left precuneus, bilateral supplementary motor areas, bilateral paracentral lobules, bilateral precentral gyri, bilateral postcentral gyri, bilateral calcarine sulci, bilateral lingual gyri, and left fusiform gyrus compared to the HC group (see Figure 1B and Table 3). Furthermore, compared to HCs, schizophrenia patients without AVHs showed reduced ALFF values in the left inferior occipital gyrus, left calcarine gyrus, and left lingual gyrus (see Figure 1C and Table 4).

**TABLE 2:** Group-level contrasts of the AVH and NAVH groups.

Cluster	Regions	Cluster size (voxels)	Peak MNI coordinate			Peak *F* values
			**X**	**Y**	**Z**	
Cluster 1	R-Frontal-Inf-Orb	20	45	−3	45	4.40

L, left; R, right; R-Frontal-Inf-Orb, right inferior frontal orbital.

**TABLE 3:** Group-level contrasts of the AVH and HC groups.

Clusters	Regions	Clusters size (voxels)	Peak MNI coordinate			Peak *F* values
			**X**	**Y**	**Z**	
Cluster 1	L-Lingual	78	−15	−57	0	−4.66
	L-Calcarine	61	−9	−75	9	−4.19
Cluster 2	R-Precentral	105	42	−15	57	−3.90
	R-Paracentral-Lobule	102	9	−33	72	−4.77
	L-Paracentral-Lobule	95	0	−21	63	−4.45
	R-Supp-Motor-Area	74	3	−15	54	−5.09
	L-Postcentral	69	−42	−24	51	−3.85
	R-Postcentral	52	36	−21	54	−4.53
	L-Precentral	44	−42	−21	51	−4.38
	L-Supp-Motor-Area	30	0	−18	60	−4.52
	L-Precuneus	29	−6	−45	66	−3.84

L, left; R, right; Supp-Motor-Area, supplementary motor area.

**TABLE 4:** Group-level contrasts of the NAVH and HC groups.

Clusters	Regions	Cluster size (voxels)	Peak MNI coordinate			Peak *F* values
			**X**	**Y**	**Z**	
Cluster 1	L-Occipital-Inf	68	−30	−78	−6	−4.35
Cluster 2	L-Lingual	117	−15	−57	0	−4.12
	L-Calcarine	68	−15	−72	6	−4.76

L-Occipital-Inf, left inferior occipital gyrus.

### Relationship to molecular architecture

**FIGURE 2:**
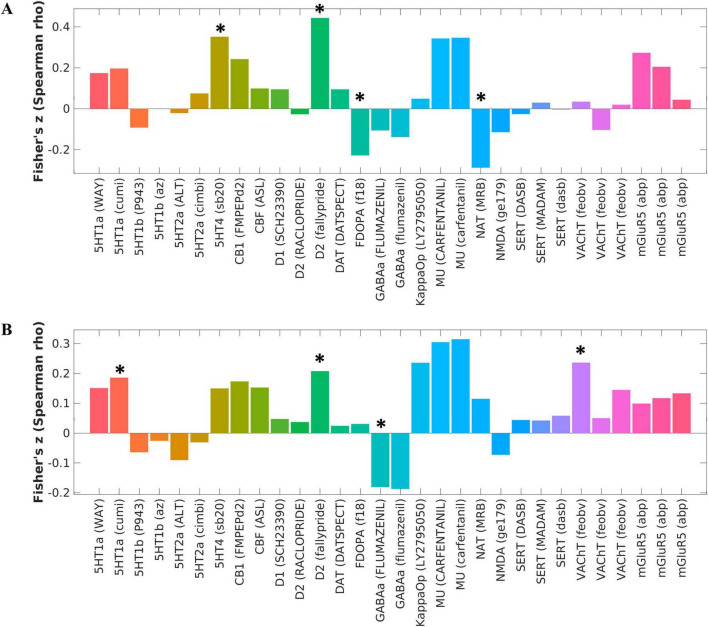
Changes in molecular structure corresponding to intergroup differences in ALFF between schizophrenia and healthy control groups. **(A)** The corresponding brain map caused by the changes of ALFF between the AVH and HC groups was significantly associated with 5-HT4, dopamine D2, F-DOPA, and NAT, as calculated by the Spearman correlation analysis. **(B)** The corresponding brain map caused by the changes of ALFF between the NAVH and HC groups was significantly associated with 5-HT1a, dopamine D2, GABAa, and VAChT, as calculated by the Spearman correlation analysis. *P < 0.05, FDR corrected.

ALFF alterations in schizophrenia patients with AVHs compared to HCs were significantly associated with 5-HT4 (*P* < 0.001, FDR corrected), dopamine D2 (*P* < 0.001, FDR corrected), F-DOPA (*P* = 0.013, FDR corrected), and NAT (*P* = 0.003, FDR corrected) (see Figure 2A and Supplementary Table 1). ALFF alterations in schizophrenia patients without AVHs compared to HCs were significantly associated with 5-HT1a: (*P* = 0.049, FDR corrected), dopamine D2 (*P* = 0.022, FDR corrected), GABAa (*P* = 0.047, FDR corrected), and VAChT: (*P* = 0.005, FDR corrected) (Figure 2B and Supplementary Table 2). There were no significant correlations between neurotransmitters and ALFF alterations in schizophrenia patients with AVHs compared with patients without AVHs.

These findings are consistent with existing research, suggesting that the dopamine system, particularly the D2 receptor, has long been associated with the positive symptoms of schizophrenia. Additionally, the 5-HT4 receptors and the norepinephrine transporter (NAT) also play important roles in emotional and cognitive regulation, potentially relating to the negative emotional content experienced during AVHs.

## Discussion

Alterations in local neural activity may signify abnormalities within specific brain regions. These functional anomalies are likely correlated with cognitive processes such as perception, attention, and emotion regulation in individuals with schizophrenia. Neurotransmitters play a crucial role in neural activity processes, exerting a significant impact on communication between neurons. In this study, we employed rs-fMRI data to investigate altered local spontaneous neural activity in schizophrenia patients with AVHs and explore the underlying molecular mechanisms of associated neurotransmitters.

### Altered ALFF patterns in schizophrenia with AVHs

In our study, we observed significant differences in local spontaneous neural activity in the brain between the AVH and NAVH groups. Notably, drug-naïve, first-episode schizophrenia patients experiencing AVHs demonstrated higher ALFF values in the right orbital part of the inferior frontal gyrus (IFG). Unlike the opercular and triangular parts of the IFG, which are part of Broca’s area (areas 44 and 45, respectively) and traditionally associated with language production and processing, the orbital part, classified as Brodmann area 47, belongs to the orbital frontal cortex (OFC) playing a critical role in emotional regulation, decision-making, and reward processing (Rolls et al., 2020; Heather Hsu et al., 2020). Previous studies have demonstrated that dysfunction of the OFC is associated with AVHs in patients with schizophrenia. Structural MRI (sMRI) findings suggest that the gray matter volume and cortical thickness of the OFC are reduced in patients with AVHs and are negatively correlated with the subscale scores for positive symptoms, including hallucinations (Takayanagi et al., 2019; Ren et al., 2024). Ren et al. (2022) found that the volume of the bilateral lateral orbitofrontal cortices was negatively correlated with the severity of persistent auditory verbal hallucinations (pAVH). Studies using positron emission tomography (PET) and functional MRI (fMRI) have also reported abnormal activation of the OFC in schizophrenia patients with AVHs (Silbersweig et al., 1995; Ait Bentaleb et al., 2006). In their exploration of brain activation patterns associated with AVHs, van Lutterveld et al. (2013) found through meta-analysis that the activation of pars orbitalis and pars opercularis in the right IFG is closely related to the generation of inner speech, without engaging cognitive processes such as stimulus detection or manual signaling. Furthermore, it is further indicated that the activation of these regions, differing from the co-activation of the insula and other regions, is specifically involved in internal speech production during the experience of AVH, rather than in tasks auditory stimulus. Currently, research on the connection between the right orbital part of the IFG and AVHs in schizophrenia is relatively scarce. Our study found a significant increase in ALFF values in the right orbital part of the IFG in patients with AVHs, suggesting that abnormal activity in this region may be related to dysfunctions in the self-monitoring system. Due to the limited number of relevant studies, the relationship between the IFG and drug-naïve, first-episode schizophrenia patients with AVHs needs to be further explored, and future research can investigate the role of different subregions of the OFC in the occurrence of hallucinations.

Furthermore, the AVH group exhibited significantly reduced ALFF compared to HCs, particularly in the default mode network areas (i.e., left precuneus) and sensorimotor network regions (i.e., bilateral supplementary motor areas, bilateral paracentral lobules, bilateral precentral gyri, and bilateral postcentral gyri). The precuneus, located on the superior parietal lobule, is an important node of the DMN. Previous studies have shown that changes in the DMN may play a key role in the development of the AVHs (Marino et al., 2022; Thomas et al., 2016; Zhuo et al., 2016). Our finding is consistent with the results of most previous studies showing lower ALFF values in the precuneus (Alonso-Solís et al., 2017; Yu et al., 2014; Hoptman et al., 2010; Xu et al., 2015) in schizophrenia patients with AVHs compared to HCs. Some research has indicated that, potentially through an altered interaction between the DMN and other networks, and/or a failure to maintain the DMN in an intrinsically stable state, internal cognition is inaccurately processed within the sensory association area, which could contribute to the AVH experience (Jardri et al., 2013; Northoff and Qin, 2011). The sensorimotor system abnormalities are important in the presence and maintenance of positive symptoms in patients with schizophrenia, especially for those with childhood onset (Berman et al., 2016; Shergill et al., 2014). A recent study found the importance of sensorimotor regions in the pathophysiology of AVH by the identified independent component (IC) from the alertness task (“temporal/sensorimotor network”) (Kubera et al., 2023). The precentral gyrus, postcentral gyrus, and paracentral lobule (the medial extension of the precentral and postcentral gyrus) are adjacent. They play a consistent role in processing and transmitting sensorimotor information, and are the main components of the sensorimotor network (Wei et al., 2018; Kaufmann et al., 2015). The precentral gyrus, which plays a role in the motor aspects of speech, is activated during the experience of AVHs (Ćurc̆ić-Blake et al., 2017b). The postcentral gyrus is an inner speech-related brain region (Moseley et al., 2013; Shergill et al., 2002; DeLisi, 2001). the decreased neural activity of the postcentral gyrus may underlie the basis for disorders of inner speech generation and imply alterations in the monitoring of inner speech, leading to a higher risk for suffering from the symptom of AVHs (Liang et al., 2022). Previous studies have found that SMA is associated with AVH in schizophrenia patients and may be involved in speech monitoring by modulating activity in auditory perception brain areas (van de Ven et al., 2009). Raij and Riekki (2012) found that schizophrenia patients with AVHs experience weaker activation in the supplementary motor area during AVHs than during imagining. However, in our investigation, discernible differences in the alterations within the default mode network and the sensorimotor network were not evident between schizophrenia patients with AVHs and those without. This finding suggests that the correlation between alterations in these networks and the specificity of AVH symptoms might be limited. Despite this, our results offer valuable insights into the neural mechanisms of auditory hallucinations in schizophrenia, suggesting an association between AVH occurrence and specific changes in brain network functions. This underscores the necessity of further exploring the unique neurobiological mechanisms of AVH, as well as the importance of investigating the interactions and overall impact of different brain networks in schizophrenia.

### Specific neurotransmitter deficits underlying ALFF alterations in schizophrenia with AVHs

To explore the underlying neurobiological mechanisms of ALFF changes observed in schizophrenia patients with AVHs, the study calculated spatial correlations between various neurotransmitter systems and ALFF alterations using JuSpace. We found that ALFF alteration patterns were significantly correlated with the dopaminergic, serotonergic and noradrenergic neurotransmitter system’s spatial distribution. On the other hand, schizophrenia patients without AVHs showed ALFF changes predominantly linked to the dopaminergic, serotonergic, (GABAa)ergic and cholinergic neurotransmitter systems, mainly involving the vesicular acetylcholine transporter. The observed significant correlation between dopamine D2 receptors and ALFF changes in schizophrenia patients, regardless of the presence of AVHs. This finding underscores the pervasive role of the dopamine system in various symptoms of schizophrenia, supporting the classical dopamine hypothesis which posits that dysfunctions in the dopaminergic system are fundamental to the pathogenesis of schizophrenia (Hietala and Syvälahti, 1996; Howes and Kapur, 2009). Notably, the correlation between dopamine D2 receptor activity and ALFF changes in patients with AVHs (*P* < 0.001) was more pronounced compared to those without AVHs (*P* = 0.022), highlighting a potential specific role of the dopamine system in AVHs (Abram et al., 2022; Mahoney et al., 2008). Additionally, compared to the healthy control group, significant changes in the dopamine precursor F-DOPA in patients with AVHs further emphasize the central role of dopamine in AVHs (Tomasella et al., 2020). Tripathi et al. (2021) demonstrated that neural circuits connecting the cortex associated with AVHs to other brain regions exhibit increased sensitivity to dopamine and neurosteroids. This heightened sensitivity disrupts normal auditory signal processing, leading patients to overreact to irrelevant sounds or misinterpret background noises and internal thoughts as external auditory stimuli, thereby triggering auditory hallucinations. Furthermore, abnormal dopamine function may disrupt these feedback mechanisms, making it difficult for patients to distinguish between internal and external auditory information, further exacerbating hallucinations. Moreover, dopamine dysregulation impairs the sensory gating function, preventing the filtering out of repetitive or irrelevant auditory stimuli and contributing to the generation of false auditory perceptions, which can also become a source of hallucinations. Additionally, we also observed significant changes in the 5-HT4 serotonin receptor and NAT in the patient group with AVHs compared to the healthy control group, changes that were not observed in patients without AVHs. The alterations in the 5-HT4 receptors may be implicated in the development of AVHs, potentially influencing emotional and cognitive processes (Hrovatin et al., 2020). Similarly, modifications in NAT levels may suggest that the noradrenaline system plays a role in the pathology of auditory verbal hallucinations (AVHs), particularly by influencing the cognitive processes in the brain related to emotional dysregulation (Mäki-Marttunen et al., 2020) as well as the interaction between the noradrenaline and dopamine systems may also represent a key mechanism underlying the occurrence of hallucinations (Ranjbar-Slamloo and Fazlali, 2020). It is noteworthy that when comparing ALFF changes between schizophrenia patients with and without AVHs, no corresponding neurotransmitter differences were observed, suggesting that the underlying neurobiological changes in schizophrenia are more complex and multifaceted. This further emphasizes the need for future research to adopt a more comprehensive approach to explore and understand these complex pathological mechanisms.

In summary, although there are currently fewer studies specifically targeting the role of the right orbital part of the IFG in AVHs, the findings of this study somewhat echo previous research on the OFC linkage to the pathophysiology of AVHs in schizophrenia and the increased sensitivity of dopamine receptors. This may offer new directions for targeted treatments. Future therapeutic strategies could consider focusing on this region. For example, repetitive transcranial magnetic stimulation (rTMS), a focal treatment, has been employed to target the area of maximal hallucinatory activation as identified in individual fMRI scans. By precisely locating and modulating brain activity in the right orbital part of the IFG, rTMS may help reduce or control symptoms of hallucinations. Additionally, individuals may be trained to decrease activity of these regions with a neurofeedback paradigm reducing the occurrence of auditory hallucinations. Simultaneously, antipsychotic medications, especially dopamine antagonists, remain the primary pharmacological treatment for hallucinations.

## Conclusion

In this study, we suggest that the JuSpace toolbox is a valuable tool for the assessment of neurotransmitter abnormalities in patients with AVHs. These patients have higher ALFF in the right orbital part of the inferior frontal gyrus (IFG), and this pattern is spatially associated with specific neurotransmitter receptors and transporters, including those in the dopaminergic, serotonergic, and noradrenergic systems. These results enhance our understanding of AVH mechanisms and their potential therapeutic targets from a neurotransmitter perspective. Future studies should consider using a broader range of biomarkers and neuroimaging techniques to more comprehensively understand the role of these neurotransmitter systems in schizophrenia, particularly in the mechanisms underlying the occurrence of hallucinations.

### Advantages and limitations

The advantages of the research are: Our study focuses on first-episode, drug-naïve schizophrenia patients, providing fresh insights into the neurobiological underpinnings of auditory verbal hallucinations (AVHs) in this specific group. The use of the JuSpace toolbox in our study has allowed for a comprehensive examination of various neurotransmitter systems, facilitating a deeper understanding of the intricate relationship between changes in ALFF and their molecular underpinnings. This approach offers insights into the altered intrinsic brain activity associated with specific neurotransmitters and enriches our knowledge of the pathophysiological processes and treatment pathways of AVHs.

The study has the following limitations: First, the relatively small sample size, predominantly first-episode, drug-naïve schizophrenia patients, may not reflect the broader population, particularly chronic cases or those in different stages of treatment.

Secondly, this study was cross-sectional. Longitudinal studies would be more informative in understanding the progression of the disease and the long-term effects of neurotransmitter changes. Thirdly, the study primarily used neuroimaging data and lacked direct biochemical or molecular data to support the observed changes in neurotransmitter systems. Future studies incorporating biochemical measures, such as cerebrospinal fluid (CSF) analysis or positron emission tomography (PET) scanning, could provide a more robust understanding of these neurochemical changes. Furthermore, while JuSpace is valuable for exploring spatial correlations between brain activity and neurotransmitter systems, it does not account for temporal dynamics of brain activity. Techniques such as electroencephalography (EEG) offer high temporal resolution and could complement JuSpace by capturing the rapid fluctuations in neural activity that are crucial for understanding transient phenomena like AVHs. By integrating EEG or other time-sensitive approaches, future research could provide a more comprehensive understanding of the spatial and temporal interactions within neurotransmitter systems and brain activity.

## Data Availability

The raw data supporting the conclusions of this article will be made available by the authors, without undue reservation.
