# Supplementary material for: Altered intrinsic neural activity and its molecular analyses in first-episode schizophrenia with auditory verbal hallucinations
Source: Front Neurosci. 2024 Oct 29;18:1478963. doi: 10.3389/fnins.2024.1478963 (PMC11554611; doi:10.3389/fnins.2024.1478963)
Supplement: Supplementary file 1 [file Table_1.docx]

**Supporting information for**

**Altered intrinsic neural activity and its molecular analyses in first-episode schizophrenia with auditory verbal hallucinations**

Table 1. Spatial correlation between ALFF and neurotransmitters map in the AVH and HC groups

| Atlas | PET Map | Fisher’s z (Spearman rho) | p-value |
| --- | --- | --- | --- |
| Default | 5-HT1a | 0.1745 | 0.16484 |
|  | 5-HT1a | 0.1966 | 0.13986 |
|  | 5-HT1b | 0.0918 | 0.53464 |
|  | 5-HT1b | 0.00031591 | 0.999 |
|  | 5-HT2a | -0.0206 | 0.81119 |
|  | 5-HT2a | 0.0747 | 0.5964 |
|  | 5-HT4 | 0.3520 | **0.000999** |
|  | CB1 | 0.2426 | 0.12787 |
|  | CBF | 0.0991 | 0.48651 |
|  | D1 | 0.0954 | 0.33367 |
|  | D2 | -0.0270 | 0.77722 |
|  | D2 | 0.4433 | **0.000999** |
|  | DAT | 0.0950 | 0.30769 |
|  | FDOPA | -0.2273 | **0.012987** |
|  | GABAa | -0.1062 | 0.26573 |
|  | GABAa | -0.1373 | 0.33666 |
|  | KappaOp | 0.0495 | 0.75624 |
|  | MU | 0.3434 | 0.058951 |
|  | MU | 0.3471 | 0.11389 |
|  | NAT | -0.2879 | **0.002997** |
|  | NMDA | -0.1139 | 0.38961 |
|  | SERT | -0.0261 | 0.77023 |
|  | SERT | 0.0300 | 0.75325 |
|  | SERT | -0.0044 | 0.96004 |
|  | VAChT | 0.0347 | 0.71029 |
|  | VAChT | -0.1038 | 0.29071 |
|  | VAChT | 0.0204 | 0.82218 |
|  | mGLUR5 | 0.2730 | 0.75924 |
|  | mGLUR5 | 0.2049 | 0.14985 |
|  | mGLUR5 | 0.0441 | 0.75325 |

Abbreviations: 5-HT1a: serotonin 5-hydroxytryptamine receptor subtypes 1a; 5-HT1b: serotonin 5-hydroxytryptamine receptor subtypes 1b; 5-HT2a: serotonin 5-hydroxytryptamine receptor subtypes 2a; 5-HT4: serotonin 5-hydroxytryptamine receptor subtypes 4; CB1: cannabinoid receptor type 1; CBF: cerebral blood flow; D1, D2, dopaminergic; DAT, dopamine transporter; F-DOPA, dopamine synthesis capacity; GABAa, gamma-aminobutric acid; KappaOp: kappa-opioid receptor; MU: mu-opioid receptor; NAT: noradrenaline transporter; NMDA: n-methyl-d-aspartate receptor; SERT: serotonin transporter; VAChT: vesicular acetylcholine transporter; mGLUR5: metabotropic glutamate receptor type 5; PET, positron emission tomography

Table 2. Spatial correlation between ALFF and neurotransmitters map in the the NAVH and HC groups

| Atlas | PET Map | Fisher’s z (Spearman rho) | p-value |
| --- | --- | --- | --- |
| Default | 5-HT1a | 0.1513 | 0.1009 |
|  | 5-HT1a | 0.1860 | **0.048951** |
|  | 5-HT1b | -0.0643 | 0.54146 |
|  | 5-HT1b | -0.0258 | 0.78921 |
|  | 5-HT2a | -0.0906 | 0.30969 |
|  | 5-HT2a | -0.0307 | 0.77922 |
|  | 5-HT4 | 0.1501 | 0.11189 |
|  | CB1 | 0.1737 | 0.11888 |
|  | CBF | 0.1530 | 0.12188 |
|  | D1 | 0.0476 | 0.63936 |
|  | D2 | 0.0372 | 0.69431 |
|  | D2 | 0.2079 | **0.021978** |
|  | DAT | 0.0249 | 0.7962 |
|  | FDOPA | 0.0310 | 0.74525 |
|  | GABAa | -0.1811 | **0.046953** |
|  | GABAa | -0.1874 | 0.063963 |
|  | KappaOp | 0.2357 | 0.14985 |
|  | MU | 0.3053 | 0.14995 |
|  | MU | 0.3150 | 0.1998 |
|  | NAT | 0.1153 | 0.20879 |
|  | NMDA | -0.0726 | 0.43057 |
|  | SERT | 0.0440 | 0.64835 |
|  | SERT | 0.0422 | 0.62937 |
|  | SERT | 0.0583 | 0.54346 |
|  | VAChT | 0.2364 | **0.004995** |
|  | VAChT | 0.0507 | 0.58941 |
|  | VAChT | 0.1447 | 0.12987 |
|  | mGLUR5 | 0.0994 | 0.32567 |
|  | mGLUR5 | 0.1176 | 0.25574 |
|  | mGLUR5 | 0.1336 | 0.1968 |

Abbreviations: 5-HT1a: serotonin 5-hydroxytryptamine receptor subtypes 1a; 5-HT1b: serotonin 5-hydroxytryptamine receptor subtypes 1b; 5-HT2a: serotonin 5-hydroxytryptamine receptor subtypes 2a; 5-HT4: serotonin 5-hydroxytryptamine receptor subtypes 4; CB1: cannabinoid receptor type 1; CBF: cerebral blood flow; D1, D2, dopaminergic; DAT, dopamine transporter; F-DOPA, dopamine synthesis capacity; GABAa, gamma-aminobutric acid; KappaOp: kappa-opioid receptor; MU: mu-opioid receptor; NAT: noradrenaline transporter; NMDA: n-methyl-d-aspartate receptor; SERT: serotonin transporter; VAChT: vesicular acetylcholine transporter; mGLUR5: metabotropic glutamate receptor type 5; PET, positron emission tomography
